# Virtual Reality as a Technological-Aided Solution to Support Communication in Persons With Neurodegenerative Diseases and Acquired Brain Injury During COVID-19 Pandemic

**DOI:** 10.3389/fpubh.2020.635426

**Published:** 2021-02-16

**Authors:** Fabrizio Stasolla, Marta Matamala-Gomez, Sara Bernini, Alessandro O. Caffò, Sara Bottiroli

**Affiliations:** ^1^“Giustino Fortunato” University of Benevento, Benevento, Italy; ^2^Department of Human Sciences for Education “Riccardo Massa”, Center for Studies in Communication Sciences “Luigi Anolli” (CESCOM), University of Milano-Bicocca, Milan, Italy; ^3^Scientific Institute for Research, Hospitalization, and Healthcare (IRCCS), Mondino Foundation, Pavia, Italy; ^4^Department of Educational Sciences, Psychology and Communication, University of Bari, Bari, Italy

**Keywords:** assistive technology, neurodegenerative diseases, healthcare, COVID19, quality of life, caregivers burden

## Abstract

The COVID-19 poses an ongoing threat to lives around the world and challenges the existing public health and medical service delivery. The lockdown or quarantine measures adopted to prevent the spread of COVID-19 has caused the interruption in ongoing care and access to medical care including to patients with existing neurological conditions. Besides the passivity, isolation, and withdrawal, patients with neurodegenerative diseases experience difficulties in communication due to a limited access to leisure opportunities and interaction with friends and relatives. The communication difficulties may exacerbate the burden on the caregivers. Therefore, assistive-technologies may be a useful strategy in mitigating challenges associated with remote communication. The current paper presents an overview of the use of assistive technologies using virtual reality and virtual body ownership in providing communication opportunities to isolated patients, during COVID-19, with neurological diseases and moderate-to-severe communication difficulties. We postulate that the assistive technologies-based intervention may improve social interactions in patients with neurodegenerative diseases and acquired brain injury-thereby reducing isolation and improving their quality of life and mental well-being.

## Introduction

Persons with neurodegenerative diseases and acquired brain injuries may fail while dealing with everyday life environmental requests. Their independence, social interactions, communication skills, and functional activities may be seriously hampered with deleterious effects on their quality of life (1–3). Isolation, passivity, and detachment may be observed with negative outcomes on caregivers and families' burden (4–6). Both intellectual and motor impairments may emerge with a significant reduction of an overall individual's functioning (7, 8). To be effective, a rehabilitative intervention should be implemented early regarding brain damage and should be intensive and assiduously prolonged over the time. Moreover, to be consolidated and generalized, the learning process should be pursued across settings (9, 10). Unfortunately, COVID-19 pandemic relevantly impeded the implementation and the realization of those conditions (11, 12). In fact, by December 24, 2020, over 72 million of cases have been documented worldwide, with almost 1.7 million of deaths (13, 14). Quarantine and social distancing interrupted public health services and regular medical care delivery (15). Public health preventions have centered around social-distancing, masks, and hand-washing strategies. Patients with chronic neurodegenerative diseases (e.g., Alzheimer, Parkinson, amyotrophic lateral sclerosis), demyelinating diseases as multiple sclerosis, and persons with acquired brain injuries (e.g., stroke, post-coma) have been impacted by lockdowns—making them vulnerable during the COVID-19 (16). In order to deal with these issues, one may envisage assistive technology (AT)-based strategies (17, 18).

AT options are broadly recognized as crucial means of support for individuals with neurodegenerative diseases and acquired brain injuries and multiple disabilities (19). Thus, AT-based programs are commonly planned to favorably fill the gap between behavioral/cognitive skills and environmental requests (20). That is, an AT setup is conceived to build functional bridges between users, environment, and technology. Essentially, it ensures that a helpful interaction (i.e., purposeful behavior and goal-oriented) is achieved for persons with extensive motor delays (21). That interaction is critical to enhancing personal fulfillment, social image, active role, satisfaction, and improving quality of life accordingly (22). Among different targeted areas, communication and leisure opportunities have been promoted (23–25). Scopus database emphasizes different empirical contributions which demonstrate the relevance and the beneficial effects on practical daily issues (e.g., communication, leisure, internet access) of technology-based programs in individuals with neurodegenerative diseases and acquired brain injuries (26–32).

At present, striking changes for public health and medical delivery services have been determined by COVID-19, including the need to find alternative solutions to take action against the substantial interruption of the regular medical care assistance and to social distance limitations forced by this pandemic (33–36). The main goal of this article is to propose a perspective on a new AT-based approach in using VR and virtual body ownership illusions to enable the communication of the patients with neurological diseases and severe-to-profound disabilities in case of isolation inside the hospital or in his/her home during this pandemic situation. In this context, current evidence-based recommendations on the use of AT-based strategies, including virtual reality (VR), as technological-aided solutions to support communication and leisure in neurological disorders, will be explored. Table 1 summarizes some relevant examples of AT-based and VR solutions for both targeted populations.

**Table 1:** Studies integrating AT-based and VR solutions to improve communication in individuals with neurodegenerative diseases and acquired brain injuries.

**References**	**Aim of the study**	**Patients (Type)**	**Intervention**	**Technology (Type)**	**Main outcomes**
**AT-based solutions**					
Lancioni et al. (17)	To study an extended smartphone-aided program that supported daily activities in addition to communication and leisure in individuals with intellectual and visual or visuo-motor disabilities	*N* = 6 participants with visual or hearing impairment. Age = between 35 and 58 years old	The participants alternated periods in which they could engage in communication and leisure with periods in which they were provided with instructions for daily activities	The extended program relied on the use of a Samsung Galaxy J4 Plus smartphone, which was fitted with Android 9.0 operating system and MacroDroid	After using the smartphone application, participants maintained successful communication and leisure engagement and started and carried out daily activities successfully
Lancioni et al. (37)	To assess an upgraded smartphone-based program to foster independent leisure and communication activity of participants with mild to moderate intellectual disability, sensory or sensory-motor impairments, and limited speech skills	*N* = 8 participants with visual or hearing impairment. Age = between 35 and 58 years old	Participants conducted leisure and communication activities by placing mini objects or pictures representing those activities and containing frequency-code labels on the smartphone. The smartphone, via the Macrodroid application, discriminated the participants' requests and provided them with the requested activities	The program was based on the use of (a) a Samsung Galaxy A3 smartphone with Android 6.0 Operating System, near-field communication, music and video player functions, and Macrodroid application, and (b) special radio frequency-code labels	After using the smartphone application, participants succeeded in requesting/accessing those activities independently and spent about 70–90% of their session time busy with those activities
Comer et al. (38)	To present an Internet-delivered Parent-Child Interaction therapy (PCIT) directly to families in their own home	Not applicable (methods article)	Not applicable (methods article)	Two computers (one in the therapist office and one in the family's home). A webcam. High-speed Internet connection	Internet-based delivery of PCIT (I-PCIT) may offer a transformative medium for overcoming traditional barriers to care. I-PCIT therapists provide remote, real-time coaching to parents during home-based parent-child interactions
Lancioni et al. (39)	To assess (a) the usability and effectiveness of the technology that allows the person to access a variety of stimulus events (e.g., songs and videos), to request caregiver's functional procedures, and to communicate with relevant partners via text messaging, and (b) the potential impact of the simultaneous availability of these technologies, in 2 post-coma persons who had emerged from a minimally conscious state and were affected by multiple disabilities	*N* = 2 participants emerged from a minimally conscious state and affected by multiple disabilities. A woman and a man of 44 and 24 years of age, respectively	The participants had to select different options within a computer program in order to access to a variety of pleasant stimuli, to request caregiver's functional procedures, and to communicate with relevant partners via text messaging. Such options were selected throughout a microswitch, which was triggered by a behavioral response and that in turn activated the choice process within the computer program	Leisure stimulus engagement and procedure requests: portable computer with commercial software, a microswitch for the participants' response, and an interface connecting the microswitch to the computer.Text messaging communication: portable computer, a GSM modem, a microswitch for the participants' response, an interface connecting the microswitch to the computer, and specifically developed software	The participants were successful at each of the three stages of the study, thus providing relevant evidence concerning performance achievements
Lancioni et al. (40)	To allow persons with multiple disabilities to (a) write and send messages to distant partners and (b) have messages from those partners read out to them	*N* = 2 women with multiple disabilities due to complications during the gestational period and to a mild form of Joubert syndrome, aged 31 and 22 years old, respectively	The participants had to select different options within a computer program in order to write and send text messages to distant partners and to have them read from such partners	A net-book computer connected to the special keyboards arranged for the two participants, a global system for mobile communication modem (GSM), a pressure microswitch to activate the computer, interfaces linking the microswitch and the GSM modem to the computer, and a specifically developed software program	The special text messaging communication system enabled the two participants to successfully communicate with distant partners, and both participants were able to write their text messages through the technology options (i.e., the special keyboards) adopted for them
**VR-based solutions**					
Isernia et al. (41)	To report results on efficiency measures and perceived functioning in real world of the Human Empowerment Aging and Disability program (HEAD), a DH-telerehabilitation system for people with chronic neurological diseases	*N* = 107 patients with Parkinson's Disease, Multiple Sclerosis, and chronic stroke. Age = between 18 and 80 years old	Patients received a 3-month rehabilitation training at home	A computer. Internet connection and motor capture devices, such as Kinect (Microsoft, WA, USA) and Leap Motion (Leap Motion Inc., CA, USA). Virtual reality scenario	The telehealth-based approach is both feasible and efficient in providing rehabilitation care to the patients from clinic to home. Increasing and maintaining participation as well as autonomy in daily routine
Baker et al. (42)	To investigate how 3D virtual environments can support social experiences in older adults	*N* = 25 older adults. Age = between 70 and 81 years old	The older adults participated in three workshops that allowed them to experience core aspects of social VR, in which they could design their own virtual avatars	Virtual avatars projected on a 180-degree semi-circular screen (workshop 2). Two Microsoft Kinect 3D cameras in each room were used to track participants' movement. Head-mounted display provided virtual embodiment (workshop 3)	The results from the workshops provide insight into older adults' design motivations when creating embodied avatars for social VR; their acceptance of social VR as a communication tool; and their views on how social VR might play a beneficial role in their lives. Participants placed critical importance on behavioral anthropomorphism—the embodied avatars' ability to speak, move, and act in a human-like manner
Slater et al. (43)	To investigate whether a body swapping to a Freud virtual body, where participants attempted to resolve their problem using their own words from two different embodied perspectives, or whether a conversation in which they talk about their problem with a pre-programmed animated virtual Freud would be equally efficacious in producing positive psychological outcomes	*N* = 69 healthy subjects. Age = between 18 and 32 years old	Participants alternating between being embodied in their own and the Freud virtual body while maintaining a self-dialogue, as if between two different people	Digital scanning set-up: iPad equipped with a structured light range sensor + inverse kinematics techniques the upper body movements of the participants could be inferred and mapped to their virtual representation. Vive head-mounted-display (HMD) made by HTC that displays a 3D scene	The results showed that the Self-Conversation method results in a greater perception of change and help compared to having a conversation with a virtual Sigmund Freud
Osimo et al. (44)	To observe how participants alternately switched between a virtual body closely resembling themselves where they described a personal problem and a VB representing Dr Sigmund Freud, from which they offered themselves counseling	*N* = 22 healthy subjects (males). Age = between 23 and 24 years old	Participants alternately switched between a virtual body closely resembling themselves where they described a personal problem and a VB representing Dr Sigmund Freud	The head-mounted display used was the Oculus DK2. Participants wore an XSens MVN motion capture suite consisting of the MVN Link 17 tracker tracking suit and MVN Studio software to stream motion data. Software Skanect (http://skanect.occipital.com/) version 1.6 to acquire the whole body scan	Counselor resembles Freud participants improve their mood, compared to the counselor being a self-representation. The improvement was greater when the Freud virtual body moved synchronously with the participant, compared to asynchronously visuo-motor coordination
Perez-Marcos et al. (45)	To propose an integrated approach that includes three key and novel factors: (a) fully immersive virtual environments, including virtual body representation and ownership; (b) multimodal interaction with remote people and virtual objects including haptic interaction; and (c) a physical representation of the patient at the hospital through embodiment agents (e.g., as a physical robot)	Not applicable (methods article)	Not applicable (methods article)	A Head-mounted display with head-tracking for immersion in the virtual environment from a first-person perspective of the avatar representing him. A wireless body tracking system for controlling over the avatar's movements. A haptic device with force-feedback is used for tactile interaction with the environment and/or remote persons. Physiological sensors and electrodes for monitoring the patients' physiological and emotional state	This unique system for telerehabilitation is the result of the integration of state of the art technologies developed at different institutions in the fields of VR, haptics, computer science, biomedical research, and neuroscience. This approach systematically differs from non-immersive telerehabilitation systems and should represent a step forward in the field

## Communication and Leisure in Neurological Disorders

Failure to positively engage in communication and leisure activities includes the incapacity to handle social interactions independently and profitably. For instance, individuals with neurodegenerative diseases and acquired brain injuries and multiple disabilities may be unable to undertake and pursue leisure activities autonomously, that is, they remain dependent on both families and caregivers. Additionally, people with neurological impairments may be unable to adequately communicate their needs or favorably make requests and choose desired items. Moreover, people with neurological impairments may face difficulties in communicating with distant partners (37, 46, 47). Technological-aided setups may be viewed as helpful supports focused on filling the existing gap between the actual individual's skills and the skill level necessary to achieve functional objectives. Accordingly, AT-based devices, setups, and tools should be rigorously customized in terms of (a) personal skills and (b) meaningful goals to be useful (48). For instance, whenever the individual has extensive motor disorders and lack of speech but is estimated within a normal range of intellectual functioning (e.g., multiple sclerosis), one may design technological aids aimed at helping the patients perform different adaptive responses. Practically, with the AT-based intervention, persons with neurodegenerative diseases and acquired brain injuries and significant impairments may be helped (a) to make small adaptive responses available in their behavioral repertoire and (b) to use adaptive responses to achieve functional tasks and/or pursue meaningful purposes (49). Communication or leisure opportunities or their combination could be embedded in a rehabilitation program using AT (39, 40, 50). Moreover, communication with distant partners through short text messages, telephone calls, or video-calls may be implemented to promote social interactions (51, 52). For instance, (53) the newly developed high-sensitivity mechanical switch for augmentative and alternative communication access in people with amyotrophic lateral sclerosis, namely the Lever Magnetic-spring Mechanical Switch (i.e., LeMMS). Results of the validation study evidenced that all the participants were capable to operate the LeMMS, which could help these patients to communicate even in an advanced stage of the disease.

## Internet-Based Communication in Neurological Disorders

Internet-based communication has revealed a useful transformative medium for overcoming traditional barriers to delivering healthcare services at patients' homes (38). Through internet-based communication services, it is possible to establish direct communication with the patients and their caregivers despite the distance. The increasing availability of internet access has already changed healthcare delivery and communication routines among patients, caregivers, and clinicians (54). Mental health difficulties are commonly documented across neurological disorders. Moreover, meta-analysis reported high anxiety and depression levels among individuals with multiple sclerosis, Parkinson's disease, and acquired brain injuries (55). Across neurological diseases, poor mental health is linked to poor quality of life, greater disability, poor prognosis and disease improvement, poor benefits after intervention (56). Accordingly, some studies have demonstrated that improving social communication in patients presenting neurodegenerative diseases and acquired brain injuries may improve their quality of life (57). Indeed, the relationship between verbal communication ability and quality of life has been shown. In detail, initial speech impairment in patients with neurodegenerative diseases and acquired brain injuries have a strong impact on their quality of life (58).

Internet connections are crucial to ensure people with timely social interactions, general public awareness, enhanced health conditions, specific knowledge on otherwise less-known neurological diseases, and health-related coping (59). Patients with neurological impairments can easily find numerous opportunities for peer social connections, learning, and leisure options (60). Furthermore, neurologists can easily manage user-generated data to satisfactory have an exhaustive representation of patients' needs and carry out epidemiological investigations (61). For example, a recent study (60) explored when and how technology could help interactions among patients with dementia and their caregivers. Three dyads patient-caregiver living in their homes were equipped with tablet computers and web-based applications and researchers analyzed their interactions. The study outlined benefits in terms of dyad interaction derived from the use of technology, suggesting the importance of an adequate provision of technology-based equipment for individuals with dementia.

Telerehabilitation (TR) offers a medium to deliver rehabilitation services and manage patients remotely using technology-based information and communication (61, 62). The adopted technologies may broadly include emails, data transmissions through videos and/or photos sent by the health provider or the user or both (63). Additionally, tablets and computers, internet-based media or programs, video conferencing, smartphones, and webinars are usually embedded (64). Typically, TR may be adopted as synchronous (i.e., the health provider and user are simultaneously connected) or as asynchronous (i.e., the health provider and user are not simultaneously connected but connected through stored data and virtual technologies or electronic communication) (41, 65). Currently, one of the newest technologies used to engage patients and caregivers in the TR training is VR systems (65, 66).

## Telepresence and Virtual Reality

At the end of the last century, VR constitutes the evolution of the old communication interfaces such as telephone, computer, and television toward the emergence of the integration of different data coming from different modalities (67). According to this, Biocca and Levy (68) defined VR as a communication system instead of a piece of technology (69). Then, VR is a communication interface connecting: (1) physical media, (2) codes, (3) information, and (4) sensorimotor channels (69). A main characteristic of VR is that allows the full immersion of the human sensorimotor channels into a vivid and realistic communication experience (68). In fact, VR is a successor of internet-based communication (70). Essentially, VR represents a technology through which it is possible to simulate existing experiences into a fake immersive virtual environment (71). Immersion is related to the extent to which the VR systems can deliver an inclusive, extensive, surrounding, and vivid illusion of reality to the participant's sensory senses. Then, immersion corresponds to the objective and quantifiable description of what the technology can provide (72). For instance, some studies demonstrated the use of VR systems, such as wearable headsets and 3-D smart televisions, to provide enjoyable, leisurely activities, with benefits in terms of quality of life, psychological well-being, and facilitated social interactions in patients with cognitive impairment (73). One example is a VR intervention using a virtual environment displayed on two large screens with head-mounted 3-D glasses and body-tracking sensors to promote engagement in patients with Mild Cognitive Impairment (74).

Another main feature of the VR system is the capability to induce a sense of “presence” into the virtual environment, which corresponds to the psychological perception of being in the virtual environment (72, 75). However, as described by Schroeder in 1996: “The notion of communications technology normally implies two or more people are involved and the emphasis is placed on the messages that pass between them” (p. 146) (76). In this regard, VR allows the possibility to interact with the immersive virtual environment and different virtual characters or avatars (77). Through VR, it is also possible to induce the sense of co-presence, that is the sense of being together in a shared space, combining significant characteristics of being both physically and socially present (78). According to this, it has been argued that the validity of the telepresence depends on the capacity to produce a context in which social actors, or social avatars in the case of VR, may communicate and cooperate between them (79–81). In line with this definition of telepresence, a large number of investigations induced telepresence within a virtual environment by means of virtual body ownership illusions (82–84). In these experimental studies, the researchers induced the illusion, by using synchronous visuo-tactile stimulation of being in the embodied virtual body instead than in the real one, inducing the sense of telepresence among the participants (82–84). Due to the possibility of moving subjects from one place to another when using VR systems, telepresence results as a promising strategy to facilitate communication with patients and their relatives when they are at home (45).

## New Communication Technologies: Virtual Reality as a Communication Tool in Clinical Populations

Based on the above-commented VR systems components, a recent study investigated how VR may contribute to older adult well-being by facilitating greater social VR participation (42, 85). In detail, in the study from Baker and colleagues, the authors conducted three workshops in which 25 older adults aged from 70 to 81 used VR as a medium for communicating with other participants (42). Older adults had to create embodied virtual avatars controlled through natural gestures and subsequently successfully and effectively used these avatars in two social VR prototypes from a third- or a first-person perspective. In this line, others used VR as a communication tool allowing medical staff to virtually interact with a virtual avatar assistant to assess and treat a virtual avatar victim presenting clinical complications (86). Additionally, virtual medical interaction with the patients through virtual avatars and telepresence has also been proposed (45, 87–89). In detail, Perez-Marcos and colleagues presented an innovative VR set-up that allows remote interaction and rehabilitation, including both the patient and doctor body projection into virtual bodies in a fully immersive environment and the physical embodiment at the remote place (45).

According to these promising studies, here we propose a VR intervention to foster and facilitate social interactions with both the clinicians and relatives with virtual avatars and telepresence in patients with neurological disorders. Such an intervention could be used when patients are isolated due to their clinical condition or to the social distance limitations forced by the COVID-19 pandemic situation. The proposed intervention aims to improve the patient's well-being avoiding his/her isolation. Specifically, it is a new VR social intervention by means of full-body ownership illusions observed from a first-person perspective and delivered through a head-mounted display and the activation of a social VR application. The effectiveness of the VR social application for enhancing social interactions through virtual avatars has been previously shown in healthy individuals (90). The intervention aims to reproduce the patients' and their relatives' body representation within the same VR environment, that is a virtual living room. In this regard, the virtual avatars' anthropomorphism characteristics are important to further enhance the sense of ownership and increase the sense of presence in interaction with other virtual avatars (42, 43, 91). This intervention is composed of five different phases: (1) the creation of the virtual avatars by scanning patients' and relatives' real bodies as in the study conducted by Osimo and co-authors or in the study by Orts-Escolano and colleagues (44, 92); (2) the integration of such virtual avatars into the VR social application; (3) delivery and teaching to use the VR social application and the head-mounted display; (4) the creation of daily social VR appointments to meet and interact with the patient's relatives during isolation or hospitalization period; and (5) the creation of weekly social VR appointments between relatives and medical staff for an updating about patient's medical condition.

The proposed intervention may represent a solution to the current worldwide situation caused by the COVID-19 pandemic, which requires suitable and effective technology-based approaches. Many recommendations are indeed proliferating about the need of alternative strategies for delivering health care services in this contingency (33, 34, 93). If some months ago the focus was mainly on the treatment of COVID-19 patients (94–99), now a days we are assisting in a growing interest for alternative ways to support the process of care of non-COVID conditions, including patients with neurodegenerative diseases and acquired brain injuries (100–102). For instance, there are many evidences about the use of telemedicine—understood as an interface in a virtual patient-clinician relationship to provide primary and secondary care (62, 99–102)—and virtual reality for remote delivery of cognitive rehabilitation in various settings of neurological care (103–105). In this frame, we propose a VR-based intervention aimed to support another critical aspect of individual's functioning that is communication, in persons with neurodegenerative diseases and acquired brain injury during COVID-19 pandemic. Thus, by using an AT-implemented strategy such as VR interventions, patients with neurodegenerative diseases may be enabled to communicate his/her needs, feelings, and thoughts to their relatives or medical staff during the hospitalization or isolation period. Accordingly, it may be fostered with positive outcomes on his/her health conditions, with a meaningful reduction of the anxiety and/or depression levels, and with beneficial effects in terms of quality of life (106, 107). Therefore, the caregivers, families, and health care systems may find a significant burden reduction (108, 109). In conclusion, through this VR intervention for communication, the patients may (a) communicate their needs, (b) independently access leisure options (e.g., positive stimulation, favorite videos, amusing songs), and (c) be connected with distant relatives. Then, patients will be more engaged in communication, and with a purposeful behavior (110, 111). In this regard, the use of new technologies for fostering engagement in patients with neurodegenerative disorders has been demonstrated (65, 112, 113).

## Concluding Remarks

The level of independence, social interactions, communication skills, and functional activities may be seriously hampered in patients with neurodegenerative diseases with deleterious effects on their quality of life (1–3). The isolation because of the COVID-19 pandemic situation may enhance such negative aspects with consequences on their quality of life (30, 114). To avoid that, some studies propose VR as a technologically-aided solution for clinical populations (99, 107, 115). However, most of these studies are focused on using VR to continue with the treatments and rehabilitation routines of the patients in case of isolation or social distancing. Depending on their level of functioning, persons with neurodegenerative diseases may be exposed to different aided-technological solutions to enhance communication skills. For example, individuals with extensive motor disabilities and moderate-to-severe intellectual disabilities may be involved in social interactions through vocal output communication aid (VOCA) or speech generating devices (SGD). Else, one may envisage hierarchical computerized systems with adapted software enabling patients with neurodegenerative diseases to request and choice desired items and/or communicate their needs. Otherwise, for individuals estimated with an intellectual normal functioning and extensive motor impairments, the independent access to the literacy through computerized systems and keyboard emulators may be proposed. Moreover, during lockdown or quarantine imposed due to an infectious disease outbreak, one may consider the communication with a distant partner through technology-aided options including a smartphone and a global system for mobile communications (GSM) system (116, 117). Here we proposed a VR-AT solution to enable the communication among patients with neurodegenerative diseases or acquired brain injury, relatives, and medical staff during the COVID-19 pandemic situation. We hypothesized that this intervention may improve the patient's social interactions enhancing their quality of life and mental well-being, avoiding isolation and negative psychological and cognitive effects.

## Data Availability Statement

The raw data supporting the conclusions of this article will be made available by the authors, without undue reservation.

## Author Contributions

FS, MM-G, and SBo have conceived the work. All authors listed have made a substantial, direct and intellectual contribution to the work, and approved it for publication.

## Conflict of Interest

The authors declare that the research was conducted in the absence of any commercial or financial relationships that could be construed as a potential conflict of interest.
